# Morphometric analysis of the inferior vena cava and its clinical correlations using abdomino-pelvic computed tomography: Series from a Jordanian population

**DOI:** 10.1016/j.ijscr.2022.107514

**Published:** 2022-08-15

**Authors:** Hadeel Odeh, Tala G. Al-Hyasat, Asil Habash, Farah J.N. Assaf, Rawand A. Sallam, Abdelbaset J. Abdellatif, Amjad Bani Hani, Darwish H. Badran, Waleed S. Mahafza, Muna A. Salameh, Amjad T. Shatarat

**Affiliations:** a Department of Basic Medical Sciences, Faculty of Medicine, Al-Balqa Applied University, Al-Salt 19117, Jordan; b King Hussein Cancer Center, Amman, Jordan; c University of Jordan, Amman, Jordan; d University Belfast, Belfast, UK; e Al-Balqa Applied University, Al-Salt, Jordan

**Keywords:** Inferior vena cava, Abdomino-pelvic computed tomography, Morphometric analysis, Diabetes mellitus, Hypertension

## Abstract

**Introduction and importance:**

This study aimed to determine the impact of DM, HTN and age on IVC dimensions as measured by CT scan relevant to guide interventions in a Jordanian population.

**Presentation of cases:**

Two hundred patients were selected from those referred to the Radiology Department, Jordan University Hospital, Amman, Jordan for clinical evaluation. Patients were divided into three age subgroups. Age, sex, and comorbidities such as DM and HTN were identified and saved for later use. All dimensions of the IVC were measured using an abdomino-pelvic CT scanner.

**Clinical discussion:**

A full morphometric analysis of the IVC would provide a better understanding of the dynamicity of the IVC in relation to its blood flow. Our results revealed that the length of the IVC was significantly shorter with age (P = 0.003). DM significantly affected the length of the IVC (P = 0.044). Hypertension also significantly affected the length of the IVC (P = 0.031), but it did not significantly affect the anterio-posterior or the transverse diameters of the IVC.

**Conclusion:**

The length of the IVC was significantly shorter with age, DM and hypertension. Morphometric measures of the IVC are of great clinical importance as they may assist in medical or surgical intervention and follow-up.

## Highlights

• Determining the main dimensions of IVC is clinically important.
• This study revealed significant variability in IVC dimensions in relation to age.
• Diabetes and hypertension are important in changing the dimensions of IVC.

## 1 Introduction

The inferior vena cava (IVC) is formed by the union of the common iliac veins at the level of the fifth lumbar vertebra [1]. It has four segments: the hepatic, suprarenal, renal, and infrarenal [2]. Being the largest vein in the human body, the IVC is considered the main venous drainage of almost all structures below the diaphragm. It has been reported that 2.6 % to 4.0 % of patients with lower limb deep vein thrombosis have IVC thrombosis, which is considered the main path for thrombus from the lower limbs to the heart [3]. Therefore, the IVC has gained considerable attention, particularly with the employment of filter insertion inside of it in patients with an absolute contraindication to anticoagulation or when there are recurrent venous thromboembolisms despite anticoagulation therapy. More than 140,000 percutaneous IVC filters have been used worldwide [4]. However, filter insertions inside the IVC were challenged by the fact that the diameter of the IVC is dynamic, and its size varies with changes in total body water and respiration [5], and its size undergoes dimensional changes in response to changes in the intravascular volume [6]. Therefore, to decrease the chances of filter malposition, preoperative computed tomography (CT)-derived measurements of the IVC were used [7], and ultrasonography-derived measurements have also been used where they demonstrated effectiveness in assessing the IVC before filter placement [8], [9]. However, a full morphometric analysis of the IVC would provide a better understanding of the dynamicity of the IVC in relation to its blood flow. Therefore, the main goal of the present study was to use CT, which is the best imaging modality for the detection of IVC pathologies and anomalies, to measure the main dimensions (length and anterio-posterior and transverse diameters) of the IVC [10]. In addition, the present study aimed to investigate the correlations between the size of the IVC and age, sex, diabetes mellitus (DM), and hypertension (HTN). This study was performed in line with PROCESS criteria [11].

## 2 Methods

This retrospective cohort study included 200 patients who were referred to Radiology Department, Jordan University Hospital, Amman, Jordan over a 4-month period. The study included consecutive cases of patients aged 20–50 years who underwent elective abdomino-pelvic CT for differential indications. The exclusion criteria were patients with history of spine surgery, renal or intraperitoneal masses, and patients with congenital anomalies or severe degenerative changes affecting the level of the vessels relative to the vertebrae. This study was approved by the institutional review board at Jordan University Hospital, 67/2019/1168, 17/3/2019. Patients were divided into three age subgroups. Age, sex, and comorbidities such as DM and HTN were identified and saved for later use.

All dimensions of the IVC were measured using an abdomino-pelvic CT scanner (128 slice; Somatom Definition, Siemens, Munich Germany) as follows: The length of the IVC was measured from the meeting point of the right and left common iliac veins to the formation of the renal vein in a coronal section. The anterior-posterior and transverse widths of the IVC were measured in an axial section at three points: pelvic (2 cm above the meeting point of the right and left common iliac veins), infrarenal (2 cm below the renal vein), and suprarenal (2 cm above the renal vein). Then, an average was obtained for the three points.

Data were collected and saved in Excel Data Sheets (version 2007, Microsoft Corp., Redmond, WA). Statistical analysis was performed using the Statistical Package for the Social Sciences version 17 (IBM Corp., Armonk, NY). The independent-samples Student's *t*-test or one-way analysis of variance followed by Dunnett's multiple comparison test was used. Statistical significance was set at p ≤ 0.05.

## 3 Results

This study included 100 men and 100 women aged 53.49 ± 15.14 years (range 19–95 years). The mean length, anterio-posterior diameter, and transverse diameter of the IVC were calculated for the entire sample according to the subgroups of age. The results are presented in Table 1. The mean length, anterio-posterior diameter, and transverse diameter of the IVC were also calculated for the whole sample 200 (100 men and 100 women) according to sex, and the results are shown in Table 2.

**Table 1:** Length and anterio-posterior and transverse diameters of the inferior vena cava in relation to age.

Age periods	20–40 years	41–60 years	60–95 years
Length	123.4 ± 2.19	118.2 ± 1.69	111.6 ± 1.88
Anterio-posterior width	53.21 ± 9.33	50.15 ± 9.81	50.50 ± 13.13
Transverse width	22.70 ± 3.81	22.79 ± 3.67	21.70 ± 3.54

**Table 2:** Mean length and anterio-posterior and transverse diameters of the inferior vena cava in relation to sex.

Diameter	Male	Female	All
Length	120.8 mm ± 13.42	113.3 mm ± 17.39	117.1 mm ± 15.97
Anterio-posterior width	17.7 mm ± 3.51	16.2 mm ± 3.63	16.9 mm ± 3.65
Transverse width	22.8 mm ± 3.6	21.9 mm ± 3.65	22.3 mm ± 3.66

Association between different dimensions of the IVC and DM.

Our data showed that the length of the IVC was significantly shorter in people with DM (113.7 ± 16.8 mm, n = 63) than in those without DM (118.6 ± 15.4 mm, n = 137; P = 0.044, *t*-test). A decrease in all diameters of the IVC was noted in people with DM. However, a non-significant statistical difference was shown in the anterio-posterior diameter of the IVC between patients with and without DM (16.5 ± 3.9 mm, n = 63 and 17.2 mm ± 3.5, n = 137, respectively; P = 0.208, *t*-test). A non-significant statistical difference was also shown in the transverse diameter of the IVC between patients with and without DM (21.9 ± 3.2 mm, n = 63 and 22.5 ± 3.8 mm, n = 137, respectively; P = 0.291, *t*-test).

Association between different dimensions of the IVC and HTN.

The results showed a significant difference in the length of the IVC between patients with and without HTN (118.9 ± 15.5 mm, n = 126 versus 113.9 ± 16.4 mm, n = 74; *P* = 0.031, *t*-test). The anterio-posterior diameter of the IVC was not statistically different in patients with and without HTN (16.8 ± 4.1 mm, n = 74 and 17 ± 3.3, n = 126; P = 0.635, *t*-test). The transverse diameter of the IVC was also not statistically different in patients with and without HTN (22.4 ± 3.3 mm, n = 74 and 22.3 ± 3.8 mm, n = 126, respectively; P = 0.798, *t*-test).

## 4 Discussion

The present study is the first to conduct a full morphometric analysis of the IVC using abdomino-pelvic CT in a Jordanian population. The length, anterio-posterior diameter, and transverse diameter of the IVC were 117.1 ± 15.97, 16.9 mm ± 3.65 mm, and 22.4 ± 3.66 mm, respectively. These dimensions were influenced by age, sex, DM, and HTN. Abdomino-pelvic CT is superior to the traditional use of cadaveric specimens to study the diameters of the IVC. In cadavers, the IVC loses its elasticity and becomes a rigid tube, which may affect its diameter compared to that in living individuals. CT imaging has been shown to be the most common imaging modality for the detection of IVC anomalies and pathologies [10]. A recent study suggested that CT should be used prior to placement of an IVC filter for better positioning [12]. However, it should be noted that CT-based studies are sometimes challenged by technical issues, which may cause some limitations. With the advent of filter insertion into the IVC, measuring the different diameters of this structure has become crucial. Different approaches have been used to measure IVC diameters for proper filter placement. For example, a radio-opaque ruler fixed behind the patient, calibrated intravascular catheter methods, and subtraction of 20 % from the measured transverse IVC diameter on radiography [13], [14]. A recent study also suggested that IVC circular geometry using cross-sectional CT should be considered in the measurement of IVC, since the IVC assumes a circular shape after the placement of the filter [13].

Abdomino-pelvic coronal section CT was performed to measure the length of the IVC. The IVC is divided into hepatic, suprarenal, renal, and infrarenal parts [2]. The hepatic part of the IVC was not measured in the present study because of technical difficulties caused by the liver. Therefore, we measured three segments of the IVC from its beginning at the meeting point of the right and left common iliac veins up to the formation of the renal vein in the coronal section.

The present study showed that the length of the IVC from its origin until the formation of the renal vein in the whole population was 117.1 mm ± 15.97. Similar results have also been reported in a different study, where the average IVC length was 106 ± 28 mm using digital subtraction angiography [15]. However, when the length of the IVC was measured by cavography, it was 96 mm, with a range between 80.3 and 142 mm [16]. These differences could be attributed to the different methodologies used in earlier studies.

The average transverse diameter of the IVC was measured from the axial section of the CT scan (22.3 mm ± 3.66). It should be noted that we measured three reference points for the transverse diameter: the pelvic (2 cm above the meeting point of right and left common iliac veins), infrarenal (2 cm below the lower renal vein), and suprarenal (2 cm above the renal vein), and then, the average of these points was calculated. Our results are in the same range as those from a study that showed that the mean IVC diameter at its largest point was 23.0 mm on CT [12].

The anterior-posterior diameter of the IVC was measured in the current study at the same reference points mentioned earlier, and the average was 16.9 mm ± 3.65. The anterio-posterior diameter of the IVC was found to be 22.3 ± 3.5 in a recent study where the cavographic vs. cross-sectional measurement of the IVC diameter before filter placement was determined. It is worth mentioning that in a later study, the anterio-posterior diameter was calculated from the infrarenal part of the IVC (4 cm) below the renal vein, where usually the IVC filter was placed [13]. Age did not affect the anterio-posterior and transverse diameters of the IVC; however, the IVC length was significantly affected by age. Unfortunately, most previous studies concentrated on the arteries when studying aging, with few articles focusing on venous aging [17]. With age, different layers of blood vessels experience structural changes (remodeling). For example, in the aorta, age has been shown to be associated with different grades of elastin fragmentation and an increase in collagen fiber deposition [18]. It has also been demonstrated that age can remodel arteries in the form of thickening or thinning of the vascular wall [19]. These changes were observed by Shatarat et al., where the diameters of the pulmonary trunk and ascending aorta were decreased or increased in different age groups [20]. Blood vessels modulate their structure under different factors, especially wall shear stress, which may cause an increase in the diameter of the blood vessel wall mass [21], and it has been demonstrated that the histologic structure of the aorta is influenced by its function; thus, the elastin/collagen ratio is higher in the ascending aorta than in the descending aorta [22]. Therefore, arteries could have different responses than veins; the IVC experiences lower pressure and shear stress than other blood vessels. Hence, it would be expected that both anterio-posterior and transverse diameters may not have been significantly affected by the remodeling process during aging in the current study. The present study did not investigate the reasons why the length of the IVC decreased with age; however, we think this is because the IVC is characterized by thicker adventitia than m.edia, which might have been altered with age, as recent studies have demonstrated that with aging, T lymphocytes invade the adventitia, and this may lead to adventitia inflammation and structural changes [23].

One of the main goals of the present study was to investigate the effects of DM on the IVC diameter. The results showed a decrease in all diameters of the IVC in patients with DM. Diabetes causes vasculopathy, leading to microvascular and macrovascular complications [24]. These complications may alter the structure of the blood vessels; for example, it has been reported that DM significantly increases the transverse diameter of the descending aorta and intraluminal diameter of the ascending aorta in diabetic women [20]. In addition, it has been demonstrated that diabetic patients have smaller aortic root diameters than non-diabetic patients [25]. It should be noted that our data showed a decrease in the transverse and anterio-posterior diameters of the IVC, which is not due to aging; hence, age in the current study did not affect these diameters. Glycemic control and duration of diabetes and how they affect the structure of veins are points to be assessed in future studies of the IVC diameter.

Another objective of the present study was to evaluate the effects of HTN on the IVC dimensions. Our results showed that there was a significant difference in the length of the IVC between patients without HTN (118.9 ± 15.5 mm) and with HTN (113.9 ± 16.4 mm), but this did not significantly affect the other dimensions. Blood vessels adapt to changes in wall shear stress due to an increase in the wall mass [21]. Pressure sensitivity can cause an increase in flow resistance when the driving pressure is increased, which can lead to structural autoregulation [26]. Thus, HTN can cause arterial stiffness [20], increased size of the abdominal aorta [27], and increased diameters of the ascending and descending aorta [20].

The present study conducted a full morphometric analysis of the IVC using abdomino-pelvic CT. However, we are limited by the source of our data, and more expanded studies are needed.

## 5 Conclusions

In conclusion, the length of the IVC was significantly shorter with age, DM and hypertension. The dimensions of the IVC are of great clinical importance as they may provide an overall picture of the expected size of the filters inserted in the IVC. In addition, morphometric analysis may assist in medical or surgical intervention and follow-up.

## Source of funding

This research did not receive any specific grant from funding agencies in the public, commercial, or not-for- profit sectors.

## Ethical approval

This study was approved by the institutional review board at Jordan University Hospital, Amman, Jordan, number 67/2019/1168, date 17/3/2019.

## Provenance and peer review

Not commissioned, externally peer-reviewed.

## Consent

Written informed consent was obtained from the patient for publication of this case report and accompanying images. A copy of the written consent is available for review by the Editor-in-Chief of this journal on request.

## Research registration

Not Applicable.

## Guarantor

HO

## CRediT authorship contribution statement

HO: Conceptualization, formal analysis, methodology, project administration, supervision, validation, visualization, writing – review and editing.

TA: Conceptualization, supervision, validation, writing – original draft preparation.

AH: Conceptualization, methodology, validation, visualization, project administration, writing – original draft preparation.

FA: Investigation, methodology, project administration, writing – review and editing.

RS: Investigation, methodology, project administration, resources, supervision, validation, visualization, writing – review and editing.

AA: Conceptualization, project administration, supervision, writing – review and editing.

AB: Investigation, methodology, project administration, supervision, writing – review and editing.

DB: Project administration, validation, writing – review and editing.

WM: Project administration, resources, validation, visualization, writing – review and editing.

MS: Project administration, validation, writing – review and editing.

AS: Conceptualization, project administration, supervision, writing – review and editing.

## Declaration of competing interest

None.
